# Data monitoring roadmap. The experience of the Italian Multiple Sclerosis and Related Disorders Register

**DOI:** 10.1007/s10072-023-06876-9

**Published:** 2023-06-14

**Authors:** Paola Mosconi, Tommaso Guerra, Pasquale Paletta, Antonio D’Ettorre, Michela Ponzio, Mario Alberto Battaglia, Maria Pia Amato, Roberto Bergamaschi, Marco Capobianco, Giancarlo Comi, Claudio Gasperini, Francesco Patti, Maura Pugliatti, Monica Ulivelli, Maria Trojano, Vito Lepore, on behalf of the Italian Multiple Sclerosis and Related Disorders Register Centres Group, U. Aguglia, MP. Amato, AL. Ancona, B. Ardito, C. Avolio, R. Balgera, P. Banfi, V. Barcella, P. Barone, P. Bellantonio, A. Berardinelli, R. Bergamaschi, P. Bertora, M. Bianchi, P. Bramanti, V. Brescia Morra, G. Brichetto, AM. Brioschi, M. Buccafusca, S. Bucello, V. Busillo, B. Calchetti, R. Cantello, M. Capobianco, F. Capone, L. Capone, D. Cargnelutti, M. Carozzi, E. Cartechini, G. Cavaletti, P. Cavalla, MG. Celani, R. Clerici, M. Clerico, E. Cocco, V. Torri Clerici, MG. Coniglio, A. Conte, F. Corea, S. Cottone, P. Crociani, F. D’Andrea, MC. Danni, G. De Luca, D. de Pascalis, M. De Riz, F. De Robertis, G. De Rosa, N. De Stefano, M. Della Corte, A. Di Sapio, R. Docimo, M. Falcini, N. Falcone, S. Fermi, E. Ferraro, MT. Ferrò, M. Fortunato, M. Foschi, A. Gajofatto, A. Gallo, P. Gallo, M. Gatto, P. Gazzola, A. Giordano, F. Granella, MG. Grasso, LME. Grimaldi, P. Iaffaldano, P. Immovilli, D. Imperiale, M. Inglese, R. Iodice, S. Leva, V. Leuzzi, A. Lugaresi, G. Lus, D. Maimone, L. Mancinelli, GT. Maniscalco, GA. Marfia, L. Margari, F. Marinelli, B. Marini, A. Marson, N. Mascoli, L. Massacesi, F. Melani, M. Merello, C. Fioretti, M. Mirabella, S. Montepietra, D. Nasuelli, P. Nicolao, L. Pasquali, F. Passantino, F. Patti, C. Pecori, M. Peresson, I. Pesci, C. Piantadosi, ML. Piras, M. Pizzorno, K. Plewnia, C. Pozzilli, A. Protti, R. Quatrale, S. Realmuto, G. Ribizzi, S. Rinalduzzi, A. Rini, S. Romano, M. Filippi, M. Ronzoni, P. Rossi, M. Rovaris, G. Salemi, G. Santangelo, M. Santangelo, A. Leone, P. Sarchielli, L. Sinisi, D. Ferraro, C. Solaro, D. Spitaleri, S. Strumia, T. Tassinari, G. Santuccio, C. Tortorella, R. Totaro, A. Tozzo, G. Trivelli, G. Turano, M. Ulivelli, P. Valentino, S. Venturi, M. Vianello, M. Zaffaroni, R. Zarbo

**Affiliations:** 1 Laboratorio di Ricerca per il Coinvolgimento dei Cittadini in Sanità, Dipartimento di Salute Pubblica, Istituto di Ricerche Farmacologiche Mario Negri IRCCS, Via Mario Negri 2, Milan, 20156 Italy; 2 Dipartimento Scienze Mediche di Base, Neuroscienze ed Organi di Senso, Università degli Studi Aldo Moro, Bari, Italy; 3 Scientific Research Area, Italian Multiple Sclerosis Foundation, Genoa, Italy; 4 Department of Physiopathology, Experimental Medicine and Public Health, University of Siena, Siena, Italy; 5 Centro Sclerosi Multipla AOU Careggi, Florence, Italy; 6 Centro Interdipartimentale Sclerosi Multipla, Fondazione Istituto Neurologico C. Mondino, Pavia, Italy; 7 Centro Sclerosi Multipla, SC Neurologia, AO Santa Croce E Carle, Cuneo, Italy; 8 Casa di Cura del Policlinico, Università Vita Salute San Raffaele, Milan, Italy; 9 UOC di Neurologia e Neurofisiopatologia Azienda Ospedaliera S. Camillo-Forlanini, Rome, Italy; 10 Centro Sclerosi Multipla AOU Policlinico Vittorio Emanuele, Catania, Italy; 11 Centro di Servizio e Ricerca sulla Sclerosi Multipla, AOU di Ferrara, Ferrara, Italy; 12 Dipartimento di Scienze Mediche Chirurgiche e Neuroscienze, Università degli Studi di Siena, Siena, Italy

**Keywords:** Register, Multiple sclerosis, Quality control, Quality indicators, Real world data

## Abstract

**Introduction:**

Over the years, disease registers have been increasingly considered a source of reliable and valuable population studies. However, the validity and reliability of data from registers may be limited by missing data, selection bias or data quality not adequately evaluated or checked.

This study reports the analysis of the consistency and completeness of the data in the Italian Multiple Sclerosis and Related Disorders Register*.*

**Methods:**

The Register collects, through a standardized Web-based Application, unique patients.

Data are exported bimonthly and evaluated to assess the updating and completeness, and to check the quality and consistency. Eight clinical indicators are evaluated.

**Results:**

The Register counts 77,628 patients registered by 126 centres. The number of centres has increased over time, as their capacity to collect patients.

The percentages of updated patients (with at least one visit in the last 24 months) have increased from 33% (enrolment period 2000–2015) to 60% (enrolment period 2016–2022). In the cohort of patients registered after 2016, there were ≥ 75% updated patients in 30% of the small centres (33), in 9% of the medium centres (11), and in all the large centres (2).

Clinical indicators show significant improvement for the active patients, expanded disability status scale every 6 months or once every 12 months, visits every 6 months, first visit within 1 year and MRI every 12 months.

**Conclusions:**

Data from disease registers provide guidance for evidence-based health policies and research, so methods and strategies ensuring their quality and reliability are crucial and have several potential applications.

**Supplementary Information:**

The online version contains supplementary material available at 10.1007/s10072-023-06876-9.

## Introduction

The use of data from disease registers has constantly grown in the past decade, providing a powerful tool to observe the course of disease and collect information about clinical practice, safety issues, research topics and patient outcomes. This has led to increased use of registers by healthcare providers and patients, demanding constant improvement of the data and procedure quality [1, 2]. Recently, the European Medicines Agency (EMA) recognized registers as important tools to support regulatory decision-making on medical products [3].

Multiple sclerosis (MS) is a chronic immune-mediated inflammatory, neurodegenerative and demyelinating disease of the central nervous system [4, 5]. Over the past decade significant progress has been made in understanding the epidemiology of the disease, and the therapeutic scenario has been expanded, allowing better management of disease course. Several guidelines have been published over the years to guide the management of persons with MS (pwMS) [6–8]. Real-world data obtained from the study of large cohorts of patients and from MS registers play an essential role, in order to outline the optimal therapeutic path. However, the legitimization of these findings must be based on accurate and standardized data collection, which calls for constant improvement and control [3, 8, 9]. A large number of MS registers have been established around the world in recent years, parallel to increasingly improved ability to collect, analyse and share huge amounts of data [10–16]. A recent survey identified 19 MS registers based in Europe [8], with the exception of the international MSBase. These routinely collected data are essential tools to provide information about epidemiological aspects, safety and treatment effectiveness, addressing and attempting to solve clinical issues in MS research. The large amount of data collected by these registers over the years forms the basis for solid and interesting population studies, such as the Post Authorization Safety Studies (PASS), in accordance with the EMA protocol on safety [17].

Between 2014 and 2015, the Italian MS Foundation in collaboration with the network of Italian MS clinical centres created the Italian MS and Related Disorders (I-MS&RD) Register, a project in continuity with the existing Italian MS Database Network set up in 2000 [12]. In line with the aim of creating an organized multicentre structure to collect data on all Italian MS patients, currently 162 centres have joined the Register, covering about 58% of the estimated 130,000 Italian pwMS [18]. Considering the huge amount of different variables collected, quality criteria need to be properly defined to encompass the entire process from data sources to register-related studies [19]. The difficulty of assessing data quality in registers stems from many factors, including the heterogeneity of research approaches and non-unified criteria for quality assessment [20]. The approach presented here is part of a broader and more transparent process of continuous improvement of the Register to fit the principles of transparency, accuracy, and completeness, and witness consistency and completeness of data collected. This study illustrates the methods and strategies about data monitoring quality developed by the I-MS&RD Register, highlighting both its importance and reliability in order to validate its epidemiological and statistical representativeness.

## Material and methods

### The Italian Register

The I-MS&RD Register officially started at the end of 2015 and is endorsed and financed by the Foundations (FISM) of the Italian MS Association (AISM), a powerful patients’ organization founded in 1968 to promote the rights of pwMS, and support a network of local branches who collaborate with healthcare professionals and clinical centres. The Register constitutes a nationwide database containing data on 77,628 (until July 2022) exclusively registered patients. An Executive Committee, jointly with a Scientific Committee, coordinate, supervise and promote all the initiatives of the Register project, a network of participant centres together with a Technical and Administrative Infrastructure (TAI) and a Technical Methodological Structure (TMS), both responsible for coordination of the activities and data management, serving as the organizational structure. A shared protocol has been developed, in order to standardize data collection and ensure high-quality data through a common platform that defines the list of variables, most with standardized options of response, together with the use of standardised data collection such as MedDRA [21], ICD-9CM [22], Eurocat [23] and FarmaDati [24]. To keep up with the protocol standards, each centre should record at least one neurological examination and an EDSS evaluation every 6 months, and an MRI every year for each patient. The Scientific Committee also agreed, by consensus, on a compulsory common minimum dataset (MDS) consisting of selected information according to principles of relevance, to ensure the collection of sufficient data for the clinical characterization of each single patient [18].

At the beginning of the project, data were stored on a client server (iMed© software), an off-line computerized medical folder. Since 2017, a web-based tool has been developed, the Web Application. The Web Application respects the standards required by the European Union General Data Protection Regulation (GDPR) 2016/679 and each centre can enter data through a secure personalized profile. Since March 2021, all the participating centres have fully adopted the Web Application. A procedures manual was also developed to facilitate consistency in protocol implementation and data collection across participants and clinical centres [25].

To boost the quality of data collection and data entry, a network of 18 research assistants (RAs) has been trained and allocated to one or more centres, depending on the centres’ contribution to the project in terms of the number of patients recorded and geographic distribution. Every year, each RA receives an activity plan with details of which centres to follow. RAs activities range from uploading new patients’ data, updating the data of registered patients, checking the quality of data according to ad hoc requests. RAs fill-in daily and monthly reports, moreover every 2 months they receive a report about the progress of new and updated patients. At least three times a year, RAs meet to discuss data collected, the centres’ involvement, or for training on new issues. According to each centre’s requests, they can be autonomous and/or they collaborate with the centre’s personnel (doctors, research nurses or data managers). The number of RAs has increased over time together with the number of centres enrolled.

### Monitoring data collection over time

This monitoring approach aims to check the progress of data collection over time and support centres’ compliance in the Register. From the 164 partner centres that signed the mandate with FISM to participate, 21 have not yet received clearance from their Ethics Committee. In the resulting 143 centres that obtained approval from the local Ethics Committee, 17 are considered “not active” due to internal issues (change of principal investigator/organizational/logistic problems). A total of 126 active centres provides patients’ data through the Web Application, so their data are eligible for this analysis.

The progress of data collection is monitored centrally bimonthly through data export. To better characterize the contribution of each centre, three convenience subgroups are considered: large (more than 1,000 patients), medium (400 to 999), and small (less than 399) centres. Considering each centre’s progress compared to the previous export, we defined as “increased” those with an increase in the number of patients, “unchanged” those with the same number of patients, “reduced” those with a drop in the number of registered patients, and “frozen” those with the same number of patients as in the five previous exports. Despite the close collaboration of the RAs with the majority of the centres (106 out of 126, until July 2022), the responsibility for accuracy and completeness of the data collected remains with the neurologists of each centre, who are regularly updated by TMS reports on the global and each centre’s specific progress and issues.

### Data checks

**Fig. 1:**
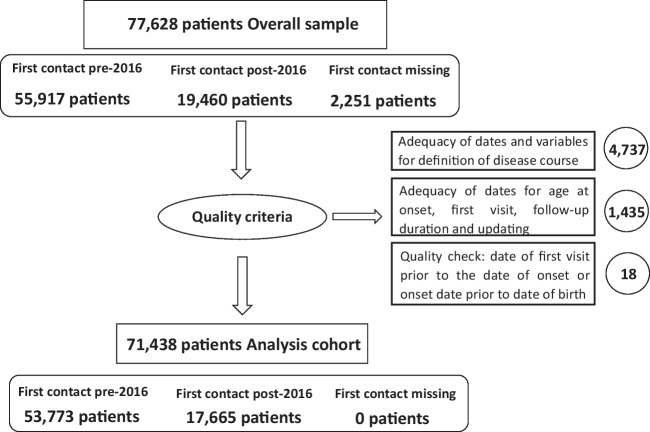
Study population

This monitoring approach aims at verifying the coherence of data collected and defining the cohort of patients eligible for the analysis (Fig. 1). The first criterion relates to the coherence of data collected, to guarantee the appropriateness and consistency of dates and variables for definition of the disease course (dates for age at onset, first visit, follow-up duration and updating). A quality check based on the exclusion of patients with the date of first visit prior to the date of onset or the date of onset prior to their date of birth, was also applied. From the “overall sample” of 77,628 registered patients, this left 71,438 patients, called “the analysis cohort”.

The second criterion can be considered a temporal cut-off, defining the cohort of patients registered prior to 1 January 2016, which included the historical cohort of patients registered since 2000, and the patients registered after this cut-off, selected as those with the earliest date between the date of the first visit and first contact at the centre. 2016 was the year of transition from the Italian MS Database Network [12] to the Register project [18]. As a consequence, there has been an expansion in the number of centres involved (from about 40 to 126), number of registered patients (from about 50,000 to 77,628), and number of variables collected (from about 400 to 1,253). In this period, the migration from the old data collection system (iMed©) to the new Web Application (now at its 3.0 release), and the RAs network was also finalised. This sub-sample counts 17,665 pwMS.

### Data quality updates

This approach aims to evaluate the updating status of data collected over time. Considering the nature of this real-world study, it is equally important to enter new data and to update those already collected, specifically in relation to new visits, therapies and disease course. For this purpose, in the Web Application, patients are classified as (i) updated, with at least one visit in the previous 2 years; (ii) recuperable, with no visit in the previous 2 years but at least one relapse, therapy or magnetic resonance imaging (MRI) recorded; (iii) lost, with no visit, relapses, therapies or MRI data recorded in the previous 2 years; (iv) undefined, with no data entered in the Register after the initial entry; (v) dropout, with data unreachable as declared by the centre; (vi) deceased (Supplementary information 1).

### Clinical indicators

These clinical indicators were created to depict the effort of the involvement of the centres not only in relation to the number of subjects recorded and their updating status, but also on a series of indicators exploring further aspects of the clinical assistance in each centre:

- Number of patients/year (the sum of each patient’s follow-up years),
- Patients with follow-up more than 5 years, sample size by centre with prospective clinical follow-up ≥ 5 years,
- Patients with active status, i.e. at least one visit and/or contact with the centre in the previous 24 months,
- A visit every 6 months,
- Expanded Disability Status Scale (EDSS) score recorded every 6 months,
- First visit within 12 months of disease onset,
- MRI (brain and spinal cord) every 12 months.

We added a new indicator, EDSS every 12 months, because of the possibility of underestimating of the score due to the COVID-19 emergency that caused the postponement of non-urgent examinations, especially among patients with a good prognosis.

Graphically, these indicators are represented as an eight-point figure, each peak representing one of the above indicators, with a scale from 1 to 5 to assess the quality scores based on quintile distribution: 5 points > 80% and ≤ 100%; 4 points > 60% and ≤ 80%; 3 points > 40% and ≤ 60%; 2 points > 20% and ≤ 40%, and 1 point > 0%; ≤ 20% [18].

In order to increase the performance of centres, a report with clinical indicators is e-mailed every 6 months to each centre where data on the all centres are reported together with ad hoc data for each centre.

We used SAS software, version 9.4 (SAS Institute, Cary, NC, USA) for all analyses.

## Results

As of July 2022, the I-MS&RD Register recorded 77,628 patients collected by 126 centres distributed across Italy. Table 1 gives an overview on centres and patients: since March 2021, the number of centres (and centres with RAs) increased, expanding the cohort of patients. Table 1 also shows increased and unchanged centres compared to the previous updates, providing an overview of their contributions. Descriptive analysis of the centres and data collected in the Register in 1 year shows a steady increase in the number of centres involved and consequently in the patient population, despite the impact of the COVID-19 pandemic on the health system and daily clinical and research work. There is an increase of increased centres — independently of their size — while the number of unchanged centres remains more stable across medium and large centres.

**Table 1:** Overview from March 2021 to July 2022 of the number of centres, RAs and patients in the Register and status of the centres in relation to their size

	Mar ‘21	May ‘21	Jul ‘21	Sep ‘21	Nov ‘21	Jan ‘22	Mar ‘22	May ‘22	Jul ‘22
Centres, RAs and patients registered									
Active Centres (No.)	112	115	117	119	120	121	122	124	126
Centres with RAs (No.)	88	99	101	93	96	101	102	105	106
Patients registered (No.)	70,493	72,283	72,959	73,564	74,417	74,931	76,088	76,883	77,628
Centre size (No., %)†									
Small Increased Unchanged	53 20 (37.7) 13 (24.5)	53 18 (33.9) 19 (35.8)	53 19 (35.8) 18 (33.9)	54 18 (33.3) 24 (44.4)	54 28 (51.9) 12 (22.2)	56 23 (41.1) 21 (37.5)	54 29 (53.7) 11 (20.4)	53 29 (54.7) 11 (20.8)	53 22 (41.5) 17 (32.1)
Medium Increased Unchanged	25 11 (44.0) 3 (12.0)	26 15 (57.7) 6 (23.1)	26 15 (57.7) 4 (15.4)	24 11 (45.8) 5 (20.8)	24 14 (58.3) 5 (20.8)	26 15 (57.7) 6 (23.1)	29 20 (68.9) 5 (17.2)	30 19 (63.3) 4 (13.3)	30 19 (70.0) 4 (20.0)
Large Increased Unchanged	23 13 (56.5) 0 (0.00)	24 14 (58.3) 2 (8.3)	24 15 (62.5) 1 (4.2)	26 16 (61.5) 4 (15.4)	26 18 (69.2) 3 (11.5)	26 17 (65.4) 3 (11.5)	26 17 (65.4) 1 (3.8)	26 21 (80.8) 1 (3.8)	26 22 (84.6) 2 (7.7)

^†^ This analysis does not include centres with ≤ 20 registered patients

**Fig. 2:**
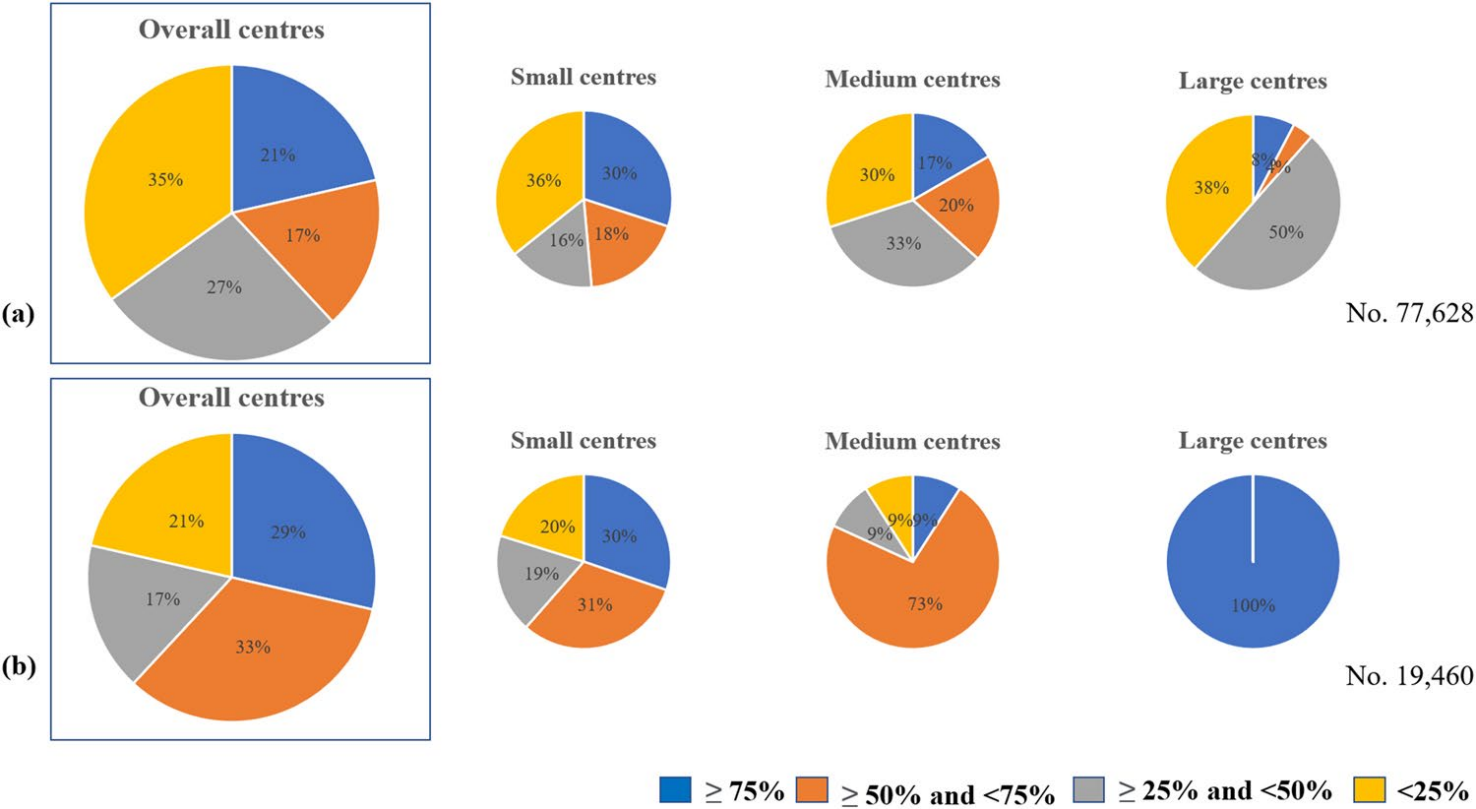
Percentages of updated† patients in the overall sample (**a**) and in the cohort of patients with the first contact after 2016 (**b**), related to centre size. A patient is considered updated when at least one of the dates about clinical visits, relapses, therapies and MRIs in the last two years is collected. † Blue indicates the centres with ≥ 75% of updated patients, orange those with ≥ 50% and < 75% of updated patients, grey those with ≥ 25% and < 50% of updated patients and yellow those with < 25%

In the overall sample, the updated patients (our gold standard) was 38.8% and its distribution varies in different periods. From 2000 to 2015, there were 18,396 updated patients on 55,917 registered patients (32.9%), while starting from 2016 there were 11,690 on 19,460 registered patients (60.1%). Figure 2 shows the contributions of updated patients. In the overall sample (a), the number of centres with ≥ 75% updated patients was 21 in small centres (30%), 5 in medium centres (17%) and 2 in large centres (8%). In the cohort of patients after 2016 (b), there were ≥ 75% updated patients in 33 small centres (30%), in (9%) 11 centres in the medium centres and 100% in the 2 large centres.

**Fig. 3:**
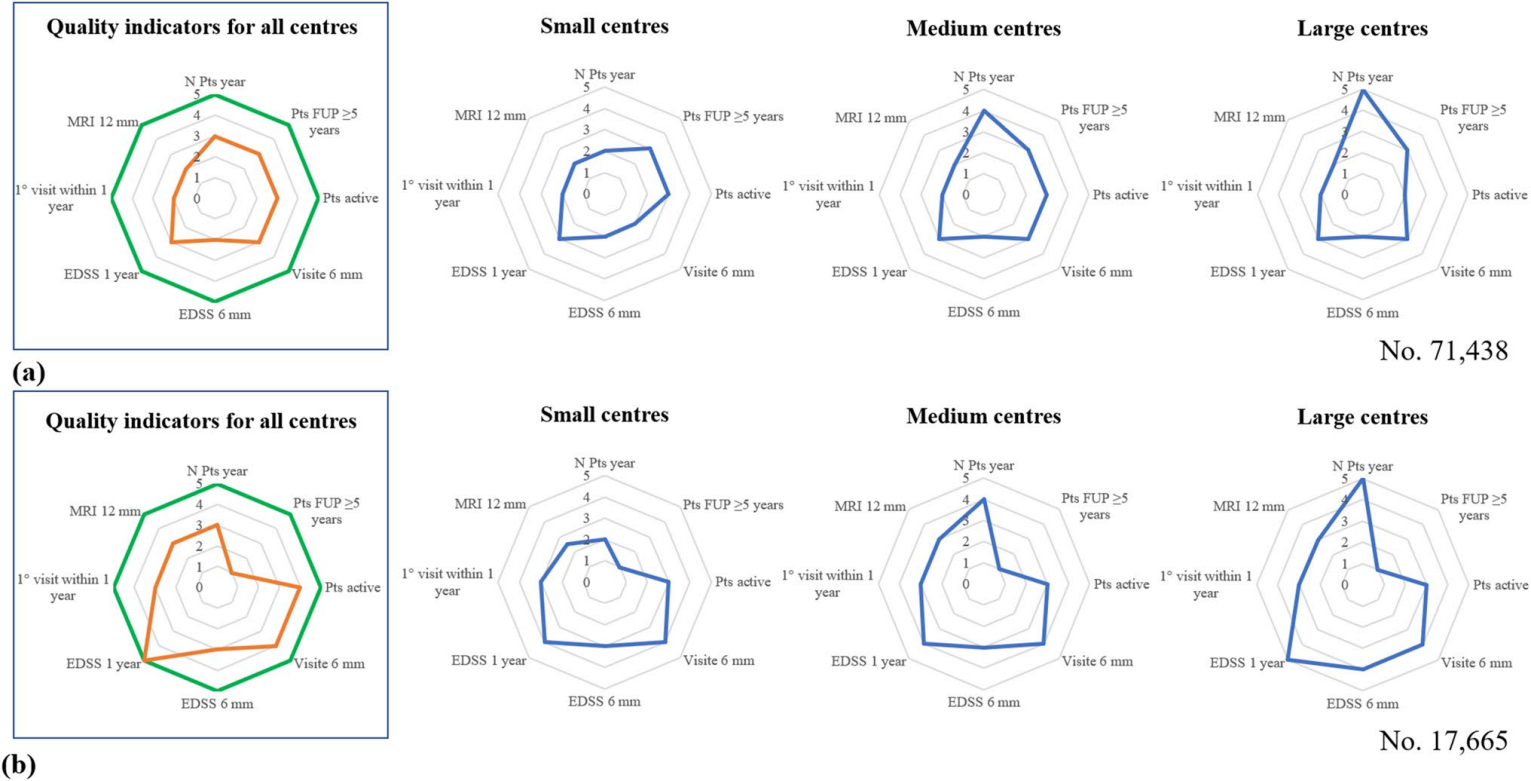
Comparison of clinical indicators for all centres and for the small, medium and large ones, (**a**) analysis cohort (**b**) cohort of patients with first contact after 2016. Green lines indicate the best possible score achievable, the orange lines indicate the actual score achieved by all centres, the blue lines the actual score achieved by small, medium and large centres

Figure 3 compares the clinical indicators between the global average and small, medium and large centres for the analysis cohort (71,438) and for the patients after 2016 (17,665). The following indicators showed significant improvement: active patients, EDSS every 6 months and every year, visits every 6 months, first visit within 1 year and MRI every 6 months. Two exceptions are the number of patients per year remaining stable, and follow-up longer than 5 years, which predictably worsens (due to its shorter follow-up period).

Table 2 shows the baseline demographics characteristics, disease type at last visit, and EDSS evaluation of the analysis cohort. In the lower part of the table are some additional data regarding selected variables collected in the Register as an example of the amount of information stored.

**Table 2:** Selection of data from the Register in the analysis cohort sample (71,438)

Demographic and clinical characteristics	
Female, No. (%)	48,079 (67.3)
Age at diagnosis (average ± SD), years Median IQR	35.7 ± 12.1 34 (26.0–43.0)
Disease duration from disease onset (average ± SD), years Median IQR	14.1 ± 10.6 12.1 (5.4–20.7)
Disease type at last visit, No. (%)	
Clinically isolated syndrome (CIS)	3842 (5.4)
Primary progressive (PP)	2616 (3.7)
Progressive-relapsing (PR)	1652 (2.3)
Relapsing–remitting (RR)	54,709 (76.8)
Secondary progressive (SP)	8386 (11.8)
EDSS	
At first visit (average ± SD)	2.4 ± 1.8
At last visit (average ± SD)	3.1 ± 2.4
At last visit ≥ 4.5 No. (%)	18,277 (25.6)
At last visit ≥ 6.0 No. (%)	10,805 (15.1)

Additional variables collected	
Periodic clinical visits, No	1,066,869
Relapses, No	225,950
Clinical events, No	37,015
MS cases treated with DMTs, No	57,642
MS cases treated with high-effective DMTs, No	24,195
EDSS evaluations, No	958,646
Cerebrospinal fluid analysis, No	32,240
MRI, No	593,701
Patients with onset under 18, No	5047
Pregnancies (from 5,789 patients), No	8851
Deceased, No	844

## Discussion

Real-world observational studies in MS, based on large clinical datasets collected in everyday practice through disease registers, offer numerous benefits for the scientific community and pwMS. Real-world data can address unanswered research questions and face and resolve the multi-faceted criticisms emerging from daily clinical practice [9, 26, 27].

In the literature, the importance of registers in MS is pointed out, supplementing randomized clinical trials data and providing fundamental information on long-term effectiveness and safety of DMTs in a real-world setting across generalizable populations, and underlining the importance of an appropriate data collection and analytical method [28]. As the MS international federation states, registers allow to put pwMS at the heart of research [29]. Studies based on registers can also be a guide in exploring potential prognostic markers of disease outcomes and in assessing effectiveness of therapies over the medium and long term, applying sophisticated statistical instruments on the large amount of data available [30].

In the constantly evolving MS research field, national and international registers and databases have developed different aims and structures over time. The I-MS&RD Register is one of the largest in Europe [8]. According to the data exports (from July 2021 to July 2022), an increase of about 5.7 new registered cases was recorded in each centre per export. The I-MS&RD Register collects about 58% of the Italian prevalence data [31]. The pwMS registered has a mean EDSS score at last visit of 3.1 showing an intermediate level of disability, but there is also a quarter of them with a significant disability (≥ 4.5) and a 15% with more severe disability (≥ 6.0), showing overall a heterogenous sample.

No data on patients reported outcomes are collected in the I-MS&RD Register; another project supported by FISM is collecting these data [32]. In order to overcome this limitation, the I-MS&RD Register working group and scientific committee are discussing the possible link from these two databases.

The validity and reliability of results from the registers may be limited by missing data, selection bias or data quality not evaluated or adequately controlled [3]. The I-MS&RD Register therefore planned a systematic analysis of the consistency, completeness and quality control of data to increase its validity and generalizability and support the compliance of centres. Along with the increasing number of centres, patients registered and updated over time, the proportion of increased centres rose, while the number of unchanged centres remained constant. Medium and small centres had higher percentages of increased cases. Large centres represent the territorial convergence of MS patients in some Italian regions, reflecting logistic difficulties in handling the large number of cases.

Since 2016, the Web Application has offered a significant improvement in what we call data quality collection: the updated cases since 2016 have increased considerably from those for 2000–2015. The percentages of updated patients have risen too, especially in medium-sized centres. This is in line with the recognition that the evolution of data collection methods with user-friendly web systems leads to highly reliable data [2].

The historical nature of the I-MS&RD Register implies greater difficulty in updating patients inserted earlier. Every 6 months, TMS updates centres on the global and each centre’s situation, clinical indicators; periodical regional meetings are organized to discuss data and possible improvement strategies. The regular monitoring of centres is leading to better data quality, demonstrated well at each data export when we constantly registered more than 8,000 updated cases in 2 months. The clinical indicators show progressive gains in data quality, particularly in the cohort of the first contact after 2016.

The network of centres is periodically encouraged by AISM and supported by a network of trained RAs with the basic aim of improving the quantity and quality of data collected. RAs play a key role in communication between TAI/TMS and each centre they are affiliated with, improving the completeness and accuracy of information shared, minimizing misunderstandings and errors.

Data quality and generalizability are closely related [11]. A recent report summarizes the results of a large systematic update and validation of the Swedish Multiple Sclerosis Register [33], noting that treatment exposure and EDSS data presented acceptable completeness but that MRI data were often missing or incomplete. The Danish Multiple Sclerosis Registry [34] guarantees completeness of data with a regular link with other registers, validity with an integrated data verification tool in the collection software, and monthly feedback to the reporting clinics on the quality indicators, and the plausibility and consistency of data within a dataset and within the longitudinal data of one patient. The MSBase, a large global MS cohort study, implemented a standardized data quality, density and generalizability process [11]. However, to our knowledge, there are still no reports that systematically monitor data in a MS register, considering the quality indicators and individual case definition parameters in the database.

The data in the I-MS&RD Register can be considered highly generalizable and reflect Italian MS patients. More than 50 research projects are now using the Register data, addressing significant research questions [35]. A reliable identification of transition to secondary progressive (SP) MS remains challenging [36]. A recent study of the I-MS&RD Register compared the data-driven SPMS definitions based on a version of Lorscheider’s algorithm and on the EXPAND trial inclusion criteria, using the neurologist’s definition as gold standard, identifying which approach had greater ability to capture SP transition [37]. Disability progression in MS is not only the result of clinical relapses, but is also secondary to Progression Independent of Relapse Activity (PIRA). A recent study from the I-MS&RD Register investigated the contribution of relapse-associated worsening and PIRA to confirmed disability accumulation in patients with clinically isolated syndrome and Relapsing Remitting MS [27]. The use of the Register data also allows to analyze and trace the path of the evolution of disability over time [38].

In 2022, the data collection platform was expanded with a new module for patients with Neuromyelitis Optica Spectrum Disorders and Myelin Oligodendrocyte Glycoprotein Antibody-associated Disease. Although they share with MS the autoimmune nature and similar clinical phenotypes, they constitute distinct entities in terms of natural history and disease characteristics. Careful collection of data for these rare diseases will allow the development of clinical and therapeutic management studies over the coming years.

Real-world data like those collected by a standardized register are valuable for evidence-based health policies and research [2, 28, 39]. Web technology, a standard coding system, and increased involvement of patients — also as a source of data [40, 41] — are contributing to the quantity and quality of data for multi-purpose potential applications [2]. The PASS studies promoted by EMA [17] as well as sharing with administrative datasets, or epidemiological studies of prognosis and outcome, are some of the advantages of registers [42, 43]. In order to gain efficient results to transfer into clinical practice, promoters, stakeholders and clinicians are aware that data need to be collected in a standardized way, through a common protocol, avoiding selection bias. Likewise, close assessment of the quality of data collected is important in order to extract meaningful findings [3, 9].

## Supplementary Information

Below is the link to the electronic supplementary material.

Supplementary file1 (DOCX 260 KB)

## Data Availability

The dataset analysed for this study is available from the corresponding author upon reasonable request.

## References

[1] Hoeijmakers F, Beck N, Wouters MWJM, Prins HA, Steup WH (2018). National quality registries: how to improve the quality of data. J Thorac Dis.

[2] Pop B, Fetica B, Blaga ML (2019). The role of medical registries, potential applications and limitations. Med Pharm Rep.

[3] EMA. Guideline on registry-based studies. European Medicines Agency. Published October 15, 2021. Accessed July 12, 2022. https://www.ema.europa.eu/en/guideline-registry-based-studies-0

[4] Harrison DM (2014) In the clinic. Multiple sclerosis. Ann Intern Med. 160(7):ITC4–2-ITC4–18; quiz ITC4–16. 10.7326/0003-4819-160-7-201404010-01004 10.7326/0003-4819-160-7-201404010-01004 24763702

[5] Lublin FD, Reingold SC, Cohen JA (2014). Defining the clinical course of multiple sclerosis. Neurology.

[6] Guidelines of the Italian Society of Neurology for diagnosis and treatment of MS [published on the Italian National Institute of Health site.

[7] Montalban X, Gold R, Thompson AJ (2018). ECTRIMS/EAN guideline on the pharmacological treatment of people with multiple sclerosis. Mult Scler Houndmills Basingstoke Engl.

[8] Ghezzi A (2018). European and American guidelines for multiple sclerosis treatment. Neurol Ther.

[9] Ezabadi SG, Sahraian MA, Maroufi H, Shahrbaf MA, Eskandarieh S (2022). Global assessment of characteristics of multiple sclerosis registries; a systematic review. Mult Scler Relat Disord.

[10] Cohen JA, Trojano M, Mowry EM, Uitdehaag BM, Reingold SC, Marrie RA (2020). Leveraging real-world data to investigate multiple sclerosis disease behavior, prognosis, and treatment. Mult Scler Houndmills Basingstoke Engl.

[11] Confavreux C, Compston DA, Hommes OR, McDonald WI, Thompson AJ (1992). EDMUS, a European database for multiple sclerosis. J Neurol Neurosurg Psychiatry.

[12] Butzkueven H, Chapman J, Cristiano E (2006). MSBase: an international, online registry and platform for collaborative outcomes research in multiple sclerosis. Mult Scler Houndmills Basingstoke Engl.

[13] Trojano M, Paolicelli D, Lepore V (2006). Italian multiple sclerosis database network. Neurol Sci Off J Ital Neurol Soc Ital Soc Clin Neurophysiol.

[14] Myhr KM, Grytten N, Aarseth JH (2012). The Norwegian Multiple Sclerosis Registry and Biobank. Acta Neurol Scand Suppl.

[15] Flachenecker P, Buckow K, Pugliatti M (2014). Multiple sclerosis registries in Europe - results of a systematic survey. Mult Scler Houndmills Basingstoke Engl.

[16] Hillert J, Stawiarz L (2015). The Swedish MS registry – clinical support tool and scientific resource. Acta Neurol Scand.

[17] Koch-Henriksen N, Magyari M, Laursen B (2015). Registers of multiple sclerosis in Denmark. Acta Neurol Scand.

[18] EMA. Patient registries. European Medicines Agency. Published September 17, 2018. Accessed July 12, 2022. https://www.ema.europa.eu/en/human-regulatory/post-authorisation/patient-registries

[19] Trojano M, Bergamaschi R, Amato MP (2019). The Italian multiple sclerosis register. Neurol Sci.

[20] Miksad RA, Abernethy AP (2018). Harnessing the power of real-world evidence (RWE): a checklist to ensure regulatory-grade data quality. Clin Pharmacol Ther.

[21] Gliklich RE, Leavy MB (2020). Assessing real-world data quality: the application of patient registry quality criteria to real-world data and real-world evidence. Ther Innov Regul Sci.

[22] MedDRA. Literature | MedDRA. Accessed July 12, 2022. https://www.meddra.org/literature

[23] Ministero della Salute. Manuale ICD-9-CM versione italiana 2007. Accessed July 13, 2022. https://www.salute.gov.it/portale/documentazione/p6_2_2_1.jsp?lingua=italiano&id=2251

[24] Eurocat. European platform on rare disease registration. Accessed July 12, 2022. https://eu-rd-platform.jrc.ec.europa.eu

[25] Farmadati. Farmadati Italia - Banca Dati del Farmaco, Parafarmaco e Dispositivo Medico. Accessed July 12, 2022. https://www.farmadati.it/

[26] AISM. SOP Manual of Procedures (MOP) for Italian multiple sclerosis register and related disorders (Version 01–15/07/2021). Accessed July 12, 2022. https://registroitalianosm.it/index.php?page=docprogetto

[27] Glaser A, Stahmann A, Meissner T (2019). Multiple sclerosis registries in Europe - an updated mapping survey. Mult Scler Relat Disord.

[28] Portaccio E, Bellinvia A, Fonderico M (2022). Progression is independent of relapse activity in early multiple sclerosis: a real-life cohort study. Brain.

[29] Ziemssen T, Hillert J, Butzkueven H (2016). The importance of collecting structured clinical information on multiple sclerosis. BMC Med.

[30] MS International Federation. Putting people with MS at the heart of research. MS International Federation. Accessed January 17, 2023. https://www.msif.org/research/challenges-of-ms-research/ms-registries-putting-people-with-ms-at-the-heart-of-research/

[31] Trojano M, Kalincik T, Iaffaldano P, Amato MP (2022). Interrogating large multiple sclerosis registries and databases: what information can be gained?. Curr Opin Neurol.

[32] AISM. Barometro sclerosi multipla Italia. Accessed July 12, 2022. https://agenda.aism.it/2022/

[33] Brichetto G, Zaratin P (2020). Measuring outcomes that matter most to people with multiple sclerosis: the role of patient-reported outcomes. Curr Opin Neurol.

[34] Alping P, Piehl F, Langer-Gould A, Frisell T (2019). Validation of the Swedish multiple sclerosis register. Epidemiol Camb Mass.

[35] Magyari M, Joensen H, Laursen B, Koch-Henriksen N (2021). The Danish multiple sclerosis registry. Brain Behav.

[36] AISM. Projects - Registro Italiano Sclerosi Multipla. Accessed July 13, 2022. https://registroitalianosm.it/index.php?page=progetticorrelati

[37] Vollmer TL, Nair KV, Williams IM, Alvarez E (2021). Multiple sclerosis phenotypes as a continuum: the role of neurologic reserve. Neurol Clin Pract.

[38] Iaffaldano P, Lucisano G, Guerra T (2022). Towards a validated definition of the clinical transition to secondary progressive multiple sclerosis: a study from the Italian MS register. Mult Scler Houndmills Basingstoke Engl.

[39] Iaffaldano P, Lucisano G, Caputo F (2021). Long-term disability trajectories in relapsing multiple sclerosis patients treated with early intensive or escalation treatment strategies. Ther Adv Neurol Disord.

[40] Concato J, Corrigan-Curay J (2022). Real-world evidence - where are we now?. N Engl J Med.

[41] Nelson EC, Dixon-Woods M, Batalden PB (2016). Patient focused registries can improve health, care, and science. BMJ.

[42] Steinemann N, Kuhle J, Calabrese P (2018). The Swiss multiple sclerosis registry (SMSR): study protocol of a participatory, nationwide registry to promote epidemiological and patient-centered MS research. BMC Neurol.

[43] Teljas C, Boström I, Marrie RA (2021). Validating the diagnosis of multiple sclerosis using Swedish administrative data in Värmland County. Acta Neurol Scand.

[44] Blaschke SJ, Ellenberger D, Flachenecker P (2022). Time to diagnosis in multiple sclerosis: Epidemiological data from the German Multiple Sclerosis Registry. Mult Scler Houndmills Basingstoke Engl.

