# Supplementary figures and images for: Data monitoring roadmap. The experience of the Italian Multiple Sclerosis and Related Disorders Register
Source: Neurol Sci. 2023 Jun 14;44(11):4001–11. doi: 10.1007/s10072-023-06876-9 (PMC10264214; doi:10.1007/s10072-023-06876-9)

**Supplementary 1** Home page screenshot of a demo centre.


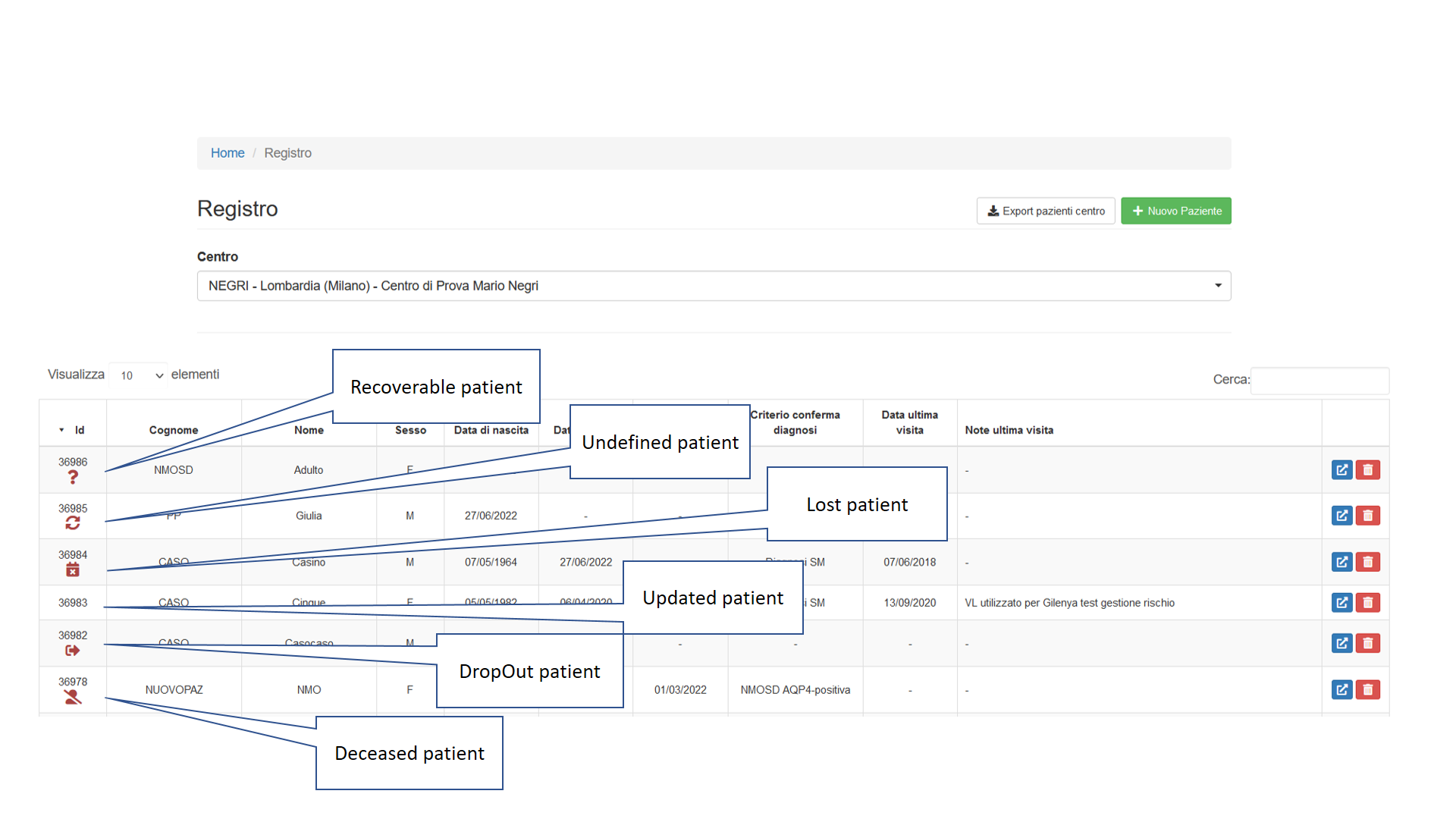

Supplement: Supplementary file 1 — Supplementary file1 (DOCX 260 KB) [file 10072_2023_6876_MOESM1_ESM.docx]
